# Alterations in the Blood Parameters and Fecal Microbiota and Metabolites during Pregnant and Lactating Stages in Bama Mini Pigs as a Model

**DOI:** 10.1155/2020/8829072

**Published:** 2020-10-26

**Authors:** Cui Ma, QianKun Gao, WangHong Zhang, Md. Abul Kalam Azad, XiangFeng Kong

**Affiliations:** ^1^CAS Key Laboratory of Agro-ecological Processes in Subtropical Region, Hunan Provincial Key Laboratory of Animal Nutritional Physiology and Metabolic Process, National Engineering Laboratory for Pollution Control and Waste Utilization in Livestock and Poultry Production, Institute of Subtropical Agriculture, Chinese Academy of Sciences, Changsha, 410125 Hunan, China; ^2^University of Chinese Academy of Sciences, Beijing 100008, China

## Abstract

This study was conducted to analyze plasma reproductive hormone and biochemical parameter changes, as well as fecal microbiota composition and metabolites in sows, at different pregnancy and lactation stages, using Bama mini pig as an experimental animal model. We found that plasma prolactin (PRL), progesterone, follicle-stimulating hormone (FSH), and estrogen levels decreased from day 45 to day 105 of pregnancy. Plasma total protein and albumin levels were lower in pregnant sows, while glucose, urea nitrogen, total cholesterol, and high-density lipoprotein-cholesterol, as well as fecal acetate, butyrate, valerate, total short-chain fatty acids, skatole, and tyramine levels, were higher in lactating sows. Interestingly, the lactating sows showed lower *α*-diversity and *Spirochaetes* and *Verrucomicrobia* relative abundances, while pregnant sows showed a higher *Proteobacteria* relative abundance. Notably, the *Akkermansia* relative abundance was highest on day 7 of lactation. Spearman analysis showed a positive correlation between plasma triglyceride and cholinesterase levels and *Akkermansia* and *Streptococcus* relative abundances. Moreover, *Oscillospira* and *Desulfovibrio* relative abundances were also positively correlated with plasma FSH, LH, and E_2_ levels, as well as PRL and LH with *Bacteroides.* Collectively, plasma reproductive hormones, biochemical parameters, and fecal microbiota composition and metabolite levels could alter along with pregnancy and lactation, which might contribute to the growth and development demands of fetuses and newborns.

## 1. Introduction

Pregnancy and lactation are extremely complex physiological processes during which a variety of systemic changes occur, including body weight, blood hormones, and fecal metabolites fluctuations, as well as immune conditions [1, 2]. Previous study showed that the host's hormones could shape the gut microbial structure and function, and the gut microbiota also altered the production and regulation of hormones in turn [3]. Thus, it is not surprising that the maternal intestinal microbiota shifts dramatically during pregancy and lactation. Accumulating evidences demonstrate that gut microbiota governs host metabolism, and its composition varies in hosts in different physiological states [4, 5]. Indeed, it has been well established that the gut microbiota composition is inconsistent in mammals during pregnancy [4, 6]. For example, the gut microbiota changes dramatically from the first to the third trimesters of pregnancy (e.g., the increased *Actinobacteria* and *Proteobacteria* relative abundances and beta diversity and reduced individual richness) [4]; the intestinal microbiota composition is also dynamic in lactating mammals [7]. However, the intestinal microbiota variation or difference in the composition during pregnancy and lactation remains poorly understood.

Notably, maternal physiological changes during pregnancy could highly influence growth and/or development of fetus, which might be influenced by gut microbes [4]. Indeed, maternal microbes (e.g., *Firmicutes* and *Proteobacteria*) could colonize the fetal/neonatal gut during pregnancy (via placenta) or lactation (via maternal milk and mother's feces), affecting the offspring's growth and development (e.g., fetal programming) [7, 8]. Because the gut microbiota is involved in regulating various host functions (e.g., nutrient absorption and body pathological or physiologic metabolism) [9] and the offspring's growth and development largely depends on maternal physiological changes during pregnancy and lactation. Therefore, it is urgent to fully understand how maternal gut microbiota composition changes and the relationship between intestinal microbiota and maternal metabolism during pregnancy and lactation.

Bama mini pig is genetically stable and size small and shares higher blood biochemical parameter, as well as internal organ shape and size similarities with humans [10]. Moreover, their gut microbiota structure and function are similar to human's [11]. Bama mini pig is the preferred experimental animal for studying how the gut microbiota changes and interacts with their host during pregnancy and lactation. Therefore, we analyzed the fecal microbiota composition and their metabolites, as well as plasma reproductive hormone and biochemical parameter levels during pregnancy and lactation. Then, correlation analyses were conducted to find the relationships between the aforementioned indexes.

## 2. Materials and Methods

### 2.1. Animals and Experimental Design

In this study, a total of 16 Bama mini pigs with 3-5 parity were herd in a mini pig farm of Goat Chong located in Shimen Town, Changde City, Hunan Province, China. After insemination, the sows were housed individually in crates (2.2 m × 0.6 m) from day 1 to day 105 of pregnancy and then housed in farrowing crates (2.2 m × 1.8 m) until weaning. Throughout the experimental period, there was no antibiotic or probiotic use, and diet intake changed with sow body condition (fed at 8:00 and 17:00 each day), and water was drunk freely. The sows were fed 0.8, 1.0, 1.2, 1.5, and 2.0 kg of the pregnant diets during 1-15, 16-30, 31-75, 76-90, and 91-105 days of pregnancy, respectively, fed 1.0 kg pregnant diets before a week of parturition and *ad libitum* after three days of parturition, and fed 2.4 kg lactation diets until weaning. The sows' nutrition met the Chinese conventional diet- (GB diet-) recommended requirements (NY-T, 2004) (Table 1).

### 2.2. Sample Collection

According to Kong et al. [12], the early, middle, and later stages of pregnancy in mini pigs are from days 1 to 45, days 46 to 75, and days 76 to delivery, respectively. During the trial period, there were four sows returned to estrus in the early pregnancy and two sows returned to estrus in the middle pregnancy. In order to minimize abortion caused by sampling stress and to ensure that the same gilts were sampled at each time point, 6-8 blood samples and feces samples of sows per stage were chosen for subsequent analysis. The litter sizes used for the experiment were 8-12.

On days 45, 75, and 105 of pregnancy and 7 and 21 of lactation, fresh sow feces were collected in 10 mL sterile centrifuge tubes and stored immediately at -20°C until processing. Meanwhile, precaval vein blood samples were collected in 10 mL heparinized tubes, centrifuged at 3500 g and 4°C for 10 min, and stored at -20°C for further analysis.

### 2.3. Determination of Plasma Reproductive Hormones and Biochemical Parameters

Plasma reproductive hormones, including prolactin (PRL), luteinizing hormones (LH), follicle-stimulating hormones (FSH), progesterone (PROG), and estradiol (E_2_), were determined using enzyme linked immunosorbent assay (ELISA) kit (Suzhou keming Co., Ltd, China), following the manufacturer's instructions.

Plasma biochemical parameters, including total protein (TP), albumin (ALB), alanine aminotransferase (ALT), aspartate aminotransferase (AST), alkaline phosphatase (ALP), urea nitrogen (UN), ammonia (AMM), glucose (GLU), triglyceride (TG), total cholesterol (TC), high-density lipoprotein-cholesterol (HDL-C), low-density lipoprotein-cholesterol (LDL-C), and cholinesterase (CHE), were determined using commercially available kits (F. Hoffmann-La Roche Ltd, Basel, Switzerland) and Roche automatic biochemical analyzer (Cobas c311, F. Hoffmann-La Roche Ltd, Basel, Switzerland).

### 2.4. DNA Extraction, PCR Amplification, and MiSeq Sequencing

Fecal microbial DNA was extracted using the Fast DNA SPIN extraction kit (MP Biomedicals, Santa Ana, CA, USA), following the manufacturer's introductions. Final DNA concentration and purity were determined using a NanoDrop ND-1000 spectrophotometer (Thermo Fisher Scientific, Waltham, MA, USA).

The bacteria 16S rRNA gene V3-V4 hypervariable region amplification was performed using the forward primer 338F (5′-GCACCTAAYTGGGYDTAAAGNG-3′) and reverse primer 806R (5′-TACNVGGGTATCTAATCC-3′) [13]. The PCR thermal cycle conditions comprised 2 min initial denaturation at 98°C; 25 cycles of 15 s at 98°C, 30 s annealing at 55°C, and 30 s elongation at 72°C; and a final extension at 72°C for 5 min. The PCR components include 5 *μ*L of Q5 reaction buffer (5×), 5 *μ*L of Q5 High-Fidelity GC buffer (5×), 0.25 *μ*L of Q5 High-Fidelity DNA Polymerase (5 U/*μ*L), 2 *μ*L (2.5 mM) of dNTPs, 1 *μ*L (10 *μ*M) each of forward and reverse primers, 2 *μ*L of DNA template, and 8.75 *μ*L of ddH_2_O. The PCR amplicons were further purified with AgencourtAMPure Beads (Beckman Coulter, Indianapolis, IN) and quantified with the PicoGreen dsDNA Assay Kit (Invitrogen, Carlsbad, CA, USA), following the manufacturer's protocols. The resulting PCR products were successfully separated using 1.2% agarose gel electrophoresis.

The equimolar purified amplicons were pooled and pair-end (2 × 300) sequenced using the MiSeq Reagent Kit v3 (600 cycles) on an Illumina MiSeq platform (Illumina, San Diego, USA), following the standard protocols by Shanghai Personal Biotechnology Co. Ltd. (Shanghai, China). Raw 16S gene data are available in the NCBI Sequence Read Archive with accession number PRJNA595474.

### 2.5. Determination of Fecal Metabolites

Fecal short-chain fatty acid (SCFA) levels were determined by gas chromatography, as previously detailed [14]. Fecal indole, skatole, and bioamine levels were determined by the reverse-phase high-performance liquid chromatography (Agilent 1290, Santa Clara, CA, USA), as previously described [14].

### 2.6. Statistical Analyses

Data analyses and graph preparation were performed using SPSS 22, Excel 2010, R package ggplot2 [15], and GraphPad Prism ver7.0 (San Diego, CA, USA). Plasma biochemical parameters, reproductive hormones, fecal metabolites, and microbiota alpha diversity were analyzed by one-way analysis of variance (ANOVA) and Duncan's multiple range post hoc test. The structural variation of microbial community among samples was analyzed with the beta diversity analysis (PERMANOVA) [16]. The relative abundance of gut microbiota at phyla and genera levels during pregnancy and lactation was analyzed via Metastats analysis (http://metastats.cbcb.umd.edu/) [17]. Spearman's correlation between the fecal metabolites, plasma indexes, and relative abundance of different microbial genera was performed using the R package [18]. All data were presented as means ± standard error of mean (SEM) and considered statistically significant when *P* < 0.05.

## 3. Results

### 3.1. Changes in Plasma Levels of Reproductive Hormone and Biochemical Parameter in Sows During Pregnancy and Lactation

**Figure 1:**
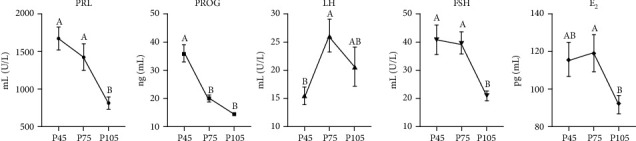
The changes of plasma reproductive hormone levels in sows at different stages of pregnancy. PRL: prolactin; PROG: progesterone; LH: luteinizing hormone; FSH: follicle-stimulating hormone; E_2_: estradiol. P45, P75, and P105 mean days 45, 75, and 105 of pregnancy, respectively. The same as below. Data show the means ± SEM. ^A,B^ indicates statistically significant differences (*P* < 0.05). *n* = 6‐8 per group.

**Figure 2:**
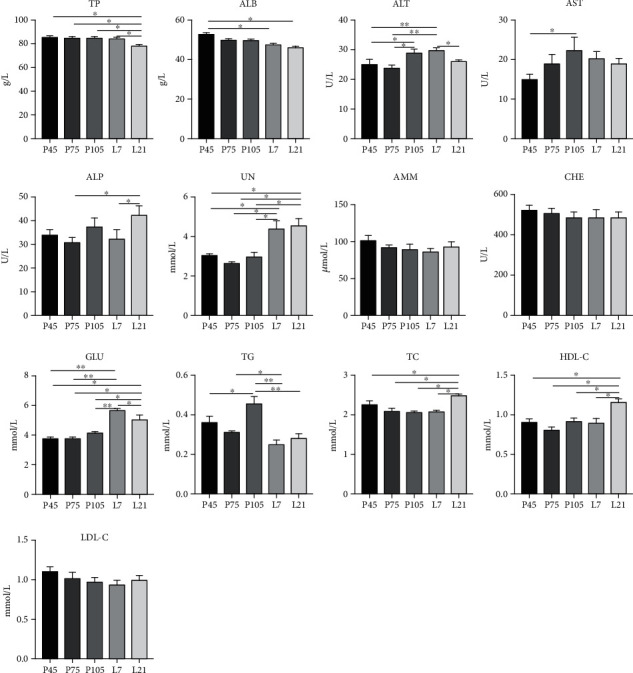
The changes of plasma biochemical parameters in sows at different stages of pregnancy and lactation. TP: total protein; ALB: albumin; ALT: alanine aminotransferase; AST: aspartate aminotransferase; ALP: alkaline phosphatase; UN: urea nitrogen; AMM: ammonia; CHE: cholinesterase; GLU: glucose; TG: triglyceride; TC: total cholesterol; HDL-C: high-density lipoprotein-cholesterol; LDL-C: low-density lipoprotein-cholesterol. L7 and L21 mean day 7 and day 21 of lactation, respectively. The same as below. Data show the means ± SEM. ^∗^*P* < 0.05, ^∗∗^*P* < 0.01. *n* = 6‐8 per group.

Plasma reproductive hormone levels changed dramatically during pregnancy (Figure 1). The PRL and FSH levels on pregnancy 105 d were significantly decreased (*P* < 0.05), compared with days 45 and 75 of pregnancy. The PROG level on days 75 and 105 of pregnancy was significantly decreased (*P* < 0.05) compared with day 45 of pregnancy. In addition, the LH level was higher (*P* < 0.05) on day 45, while E_2_ level was lower (*P* < 0.05) on day 105, compared with the day 75 of pregnancy. The plasma levels of biochemical parameters associated with protein and gluco-lipid metabolism in sows also changed differently from pregnancy to lactation (Figure 2). Plasma TP and ALB levels were significantly lower (*P* < 0.05) in all pregnant stages, whereas UN and GLU were higher (*P* < 0.05) during lactation. Notably, ALP activity on lactation 21 d was higher (*P* < 0.05) than on pregnancy 75 d and lactation 7 d. Both AST and ALT activities on pregnancy 105 d were higher (*P* < 0.05) than on 45 d. In addition, plasma biochemical parameters associated with nitrogen metabolism changed with pregnancy and lactation. On lactation 21 d, TC and HDL-C levels were higher (*P* < 0.05) than those of other pregnant stages and the TG level highest (*P* < 0.05) on pregnancy 105 d.

### 3.2. The Fecal Microbiota Community Profile in Sows during Pregnancy and Lactation

**Figure 3:**
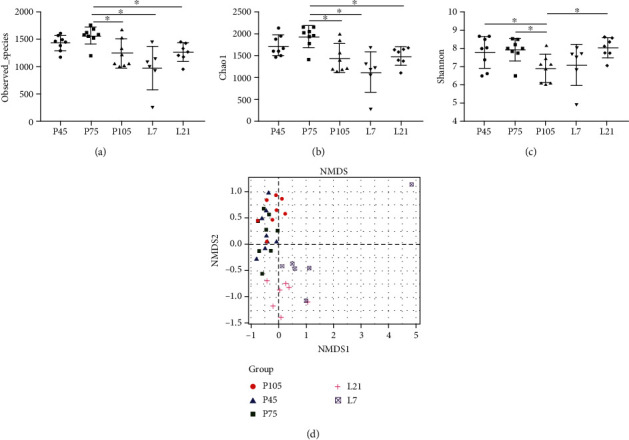
Fecal microbiota diversity of sows at different stages of pregnancy and lactation. The bacterial diversity was estimated by observed_species (a), Chao1 (b), Shannon index (c), and nonmetric multidimensional scaling (NMDS) of bacterial community (d). Data show the means ± SEM. *n* = 6‐8 per group.

A total of 1,465,181 sequences were obtained from 37 samples after size filtering, quality control, and chimera removal, with an average of 39,600 sequences per fecal sample. Based on a 97% similarity, 48,615 operational taxonomic units (OTUs) were obtained with an average of 1,314 OTUs per sample (Supplementary Table S1). The diversity and richness of the observed_species of fecal microbiota in gestating and lactating sows were measured by the Chao1 and Shannon indexes, respectively (Figure 3). Observed_species between pregnancy and lactation were significantly different, with highest Chao1 index (*P* < 0.05) on pregnancy 75 d. However, the Shannon index on pregnancy 105 d was lowest (*P* < 0.05) among the different stages.

Nonmetric multidimensional scaling (NMDS) can assess the between group distance in the sow fecal bacteria community structure. Microbial community profiles were clustered more closely to each other from pregnancy days 45 to 105, while clearly separated between gestation and lactation (Figure 3(d)), indicating that the *β*-bacterial diversity of samples between gestation and lactation differed significantly.

### 3.3. Change of Fecal Microbial Community and Composition in Sows During Pregnancy and Lactation

**Figure 4:**
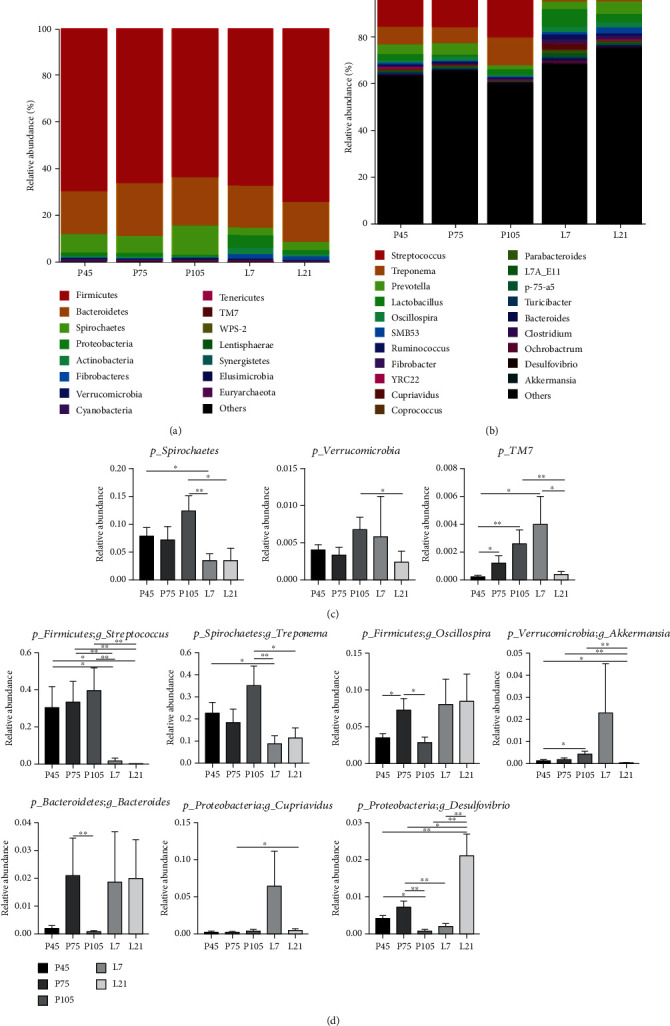
Fecal microbiota community structure of sows at different stages of pregnancy and lactation. Fecal microbiota distributed at phylum (a) and genera (b) levels. All of the phyla were listed, and only the top twenty genera were listed. Comparison of relative abundances at the phylum (c) and genera (d) levels, and discrepancy of the top ten fecal microbiota was listed. *n* = 6‐8 per group.

The microbial community compositions of all fecal samples were analyzed at phylum and genus levels. Based on 97% 16S rRNA gene sequence identity, 15 microbial phyla and top 20 microbial genera, with a clear classification status, were identified in all pregnancy and lactation stages. Firmicutes (69.58%, 66.15%, 63.69%, and 74%), Bacteroidetes (18.4%, 22.75%, 20.71%, and 17.1%), and Spirochaetes (7.88%, 7.13%, 12.40%, and 3.74%) were the top three dominant phyla on pregnancy 45, 75, and 105 d and on lactation 21 d, respectively; whereas Firmicutes (67.11%), Bacteroidetes (18.08%), and Proteobacteria (5.17%) were the top three dominant phyla on lactation 7 d (Figure 4(a)). Aside from these dominant phyla, there were other dominant phyla with >1% relative abundances of the total microbial composition, including Proteobacteria (1.39%), on pregnancy 45 d; Proteobacteria (1.27%), 105 d; Spirochaetes (3.27%), Actinobacteria (2.85%), and Fibrobacteres (1.85%) on lactation 7 d; and Fibrobacteres (1.54%), Proteobacteria (1.39%), and Actinobacteria (1.17%) on lactation 21 d (Figure 4(a)). At the genus level, 20 bacterial taxa distributions were clearly and visually presented (Figure 4(b)), indicating the varied relative abundances of bacterial genera from pregnancy to lactation, with the top three dominant genera, including Streptococcus (9.66%), Treponema (6.75%), and Prevotella (3.86%).

### 3.4. Differences in Microbial Communities during Pregnancy and Lactation

The different fecal microbial phylum abundances between pregnancy and lactation are shown in Figure 4(c). Verrucomicrobia and Spirochaetes relative abundances increased (*P* < 0.05) during pregnancy but decreased (*P* < 0.05) during lactation. Further, TM7 relative abundance was the highest (*P* < 0.05) on lactation 7 d among all the groups.

Fecal microbial relative abundance at the genus level varied greatly among different stages of pregnancy and lactation (Figure 4(d)). The relative abundances of *Streptococcus* and *Treponema* during lactation were significantly lower (*P* < 0.05) than those during pregnancy. Interestingly, *Akkermansia*, *Bacteroides*, and *Desulfovibrio* relative abundances were higher (*P* < 0.05) during lactation than pregnancy.

### 3.5. Changes in Fecal Metabolite Abundances in Sows During Pregnancy and Lactation

**Figure 5:**
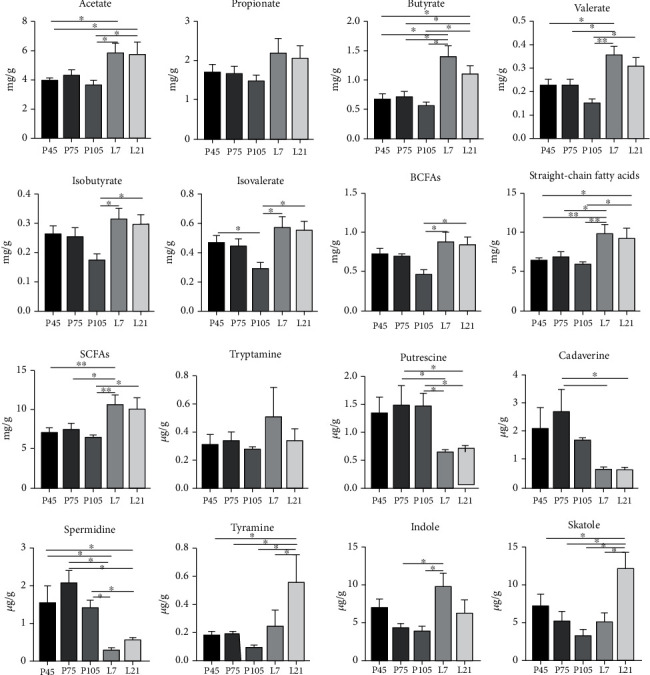
The levels of short-chain fatty acids (SCFAs), bioamines, indole, and skatole of sows at different stages of pregnancy and lactation. BCFAs: branched-chain fatty acids. Data show the means ± SEM. *n* = 6‐8 per group.

The changes in sow fecal metabolites during pregnancy and lactation are shown in Figure 5. Acetate, propionate, butyrate, valerate, straight-chain fatty acids, and SCFAs levels were higher (*P* < 0.05) during lactation than pregnancy. Interestingly, isobutyrate, isovalerate, and branched-chain fatty acids (BCFAs) levels on day 105 of pregnancy, were the lowest (*P* < 0.05) compared to other stages. Putrescine, cadaverine, and spermidine levels were lower (*P* < 0.05) during lactation, whereas tyramine, indole, and skatole levels were higher (*P* < 0.05), compared with those during pregnancy.

### 3.6. Correlation between Fecal Microbiota Relative Abundances and Plasma Parameters

**Figure 6:**
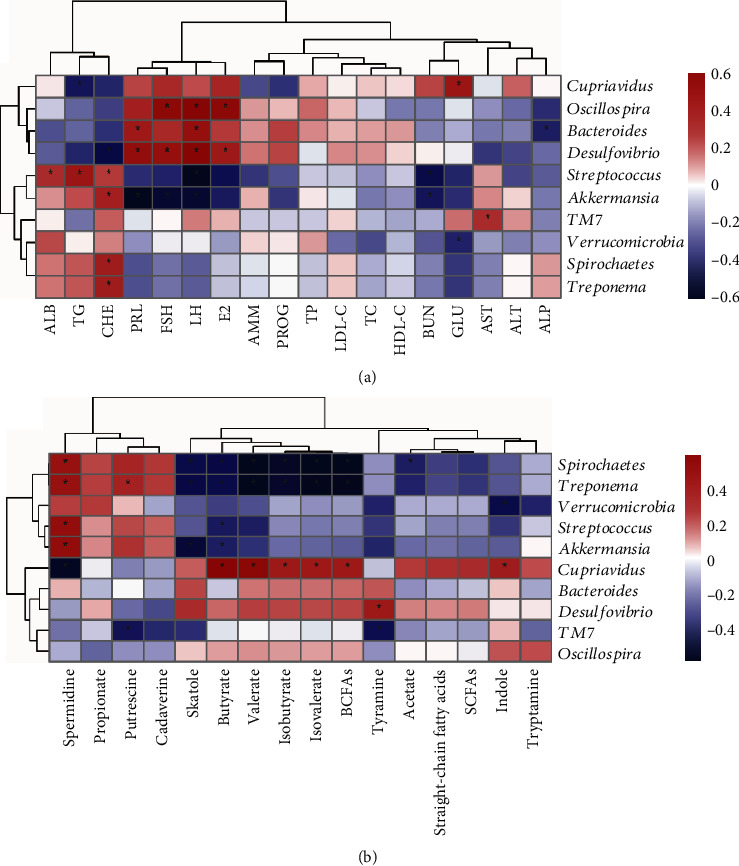
Correlations between the discrepant fecal microbiota and metabolite levels of sows. The plasma parameters (a) and fecal metabolites (b) are presented to be correlated with gut microbiota, respectively. Spearman correlations were used, and ^∗^ means the correlation significant. PRL: prolactin; LH: luteinizing hormone; FSH: follicle-stimulating hormone; PROG: progesterone; E_2_: estradiol; TP: total protein; ALB: albumin; ALT: alanine aminotransferase; AST: aspartate aminotransferase; ALP: alkaline phosphatase; UN: urea nitrogen; AMM: ammonia; GLU: glucose; TG: triglyceride; TC: total cholesterol; HDL-C: high-density lipoprotein-cholesterol; LDL-C: low-density lipoprotein-cholesterol; CHE: cholinesterase; BCFAs: branched-chain fatty acids; SCFAs: short-chain fatty acids.

The correlation between fecal microbiota and plasma reproductive hormones or biochemical parameters is presented in Figure 6(a). There were positive correlations (*P* < 0.05) between microbial relative abundances and plasma indexes levels, including Cupriavidus, Proteobacteria, and Oscillospira and E_2_, LH, and FSH; Bacteroides and LH and PRL; Desulfovibrio and E_2_, LH, FSH, and PRL; Streptococcus and CHE, TG, and ALB; and Akkermansia and CHE. There were negative correlations (*P* < 0.05) between Bacteroides and ALP; Desulfovibrio and CHE; Streptococcus and UN and LH; Akkermansia and LH, FSH, and PRL; and Verrucomicrobia and GLU.

### 3.7. Correlation between Fecal Metabolite Levels and the Relative Abundances

The Spearman correlation between the fecal metabolites and microbial genera abundances in the sows is shown in Figure 6(b). The relative abundances of Spirochaetes and Treponema were negatively correlated with valerate, butyrate, BCFAs, isovalerate, isobutyrate, and skatole levels and were positively with spermidine level (*P* < 0.05). Moreover, Spirochaetes was negatively correlated (*P* < 0.05) with acetate level, but Treponema was positively correlated (*P* < 0.05) with putrescine level. Verrucomicrobia was negatively correlated (*P* < 0.05) with indole level; Streptococcus was negatively with butyrate level and positively (*P* < 0.05) with spermidine level; Akkermansia was negatively (*P* < 0.05) with butyrate and skatole levels and positively (*P* < 0.05) with spermidine level; Cupriavidus was positively (*P* < 0.05) with valerate, butyrate, BCFAs, isovalerate, isobutyrate, and indole levels and negatively (*P* < 0.05) with spermidine level; and Desulfovibrio was positively (*P* < 0.05) with tyramine level.

## 4. Discussion

Many intricate and comprehensive physiology and metabolism alterations occur in sows during pregnancy and lactation, accompanied with hormonal and metabolic changes to meet maternal nutrient needs and support growth and development of offspring. In this study, changes in fecal microbiota composition and metabolites, as well as protein and gluco-lipid metabolisms, were analyzed at pregnancy (from day 45 to day 105) to lactation (from day 7 to day 21) stages. Our data showed that maternal fecal microbiota composition and metabolite alterations changed during pregnancy and lactation to meet the growth and development demands of their fetuses and/or newborns.

The nutrient metabolism status of sows during pregnancy and lactation are crucial for modulating body health and offspring growth. Generally, plasma TP and ALB levels reflect the absorption and metabolism of protein in sows [19]. Plasma UN level can evaluate the body protein and amino acid catabolism status, and a decrease in plasma UN level reflects that the amino acid composition in body is well balanced [20]. In this study, plasma TP and ALB levels were higher, and the UN level was lower during pregnancy stage than lactation, implying that protein utilization was increased in sows during pregnancy, which could benefit sows to favor growth and development of their fetuses. The higher plasma UN level during lactation might be induced by higher crude protein (16.30%) level in lactating diets. The lactating sows were fed 2.4 kg diets with a 16.30% CP level while the pregnant sows were fed 0.8-2.0 kg diets with 12.82% CP level. The higher plasma UN level might cause an increase of milk UN level, which is positively correlated with blood UN level [21], suggesting that it can facilitate fat deposition and growth and development of suckling piglets [22].

Blood TG, TC, HDL-C, and LDL-C levels reflect the lipid utilization and absorption [12]. HDL-C can transport TC to the liver for metabolizing to be other substances, thereby maintaining a stable TC level in the body [23]. The plasma TC and HDL-C levels were higher on day 21 of lactation than other stages in the present study, consistent with a previous report [6], suggesting that lipoprotein metabolism was enhanced in sows to offset the nutrient loss induced by lactation. Previous studies demonstrated that there is a diabetogenic status in late pregnancy, including high TG level, insulin resistance, and/or hyperglycemia, which could provide adequate nutrients to the fetuses, as well as fulfill the energy demands of infants during lactation [24, 25]. Koren found that the reduced insulin sensitivity and increased blood glucose level in pregnant women are beneficial in the context of a normal pregnancy [4]. Interestingly, we found that plasma TG level was the highest on day 105 of pregnancy, while GLU level presented an increasing trend during pregnancy, suggesting that metabolic syndrome in sows is existed during late pregnancy to favor the availability of glucose and other nutrients for growth and development of fetuses.

Reproductive hormones change regularly during the reproductive cycle. FSH can promote follicular development and act synergistically with LH to promote the secretion of E_2_ by host, which roughly complied with our findings that a synergic relationship between FSH, LH, and E_2_. Additionally, proliferation of some bacterial species (e.g., *Lactobacillus*, *Streptococcus*, and *Escherichia coli*) was enhanced by female hormones [26]. There was a similar changed trend between PROG, E_2_, and PRL during different pregnancy stages, which were the lowest on pregnancy 105 d. Indeed, several bacteria (e.g., *Bacteroides*, *unclassified Lachnospiraceae*, *Clostridiales*, and *Akkermansia*) could metabolize E_2_ and PROG [27], suggesting that there is an association between hormones and gut microbiota. Moreover, Adlercreutz reported that there was a correlation between E_2_ level and fecal microbiota composition and abundance [28], and Kornman and Loesche found E_2_ and PROG could foster the growth of *Bacteroides* (including *Prevotella intermedius*) by taking up E_2_ and PROG [27]. The mechanism might be that gut microbiota composition was affected by the changes of hormones receptor [29], and some bacteria have also been found to involve in the secretion or modification of hormone [30]. SCFAs (including acetate, propionate, and butyrate) produced by gut microbiota could inhibit PRL gene transcription, thus reducing the PRL production [31]. These findings suggested that gut microbiota played an important role in hormones metabolism of host.

Gut microbiota structure and composition shift dramatically during pregnancy and lactation. The alpha diversity of gut microbiota is closely related to host health [32], and low alpha diversity is expected to mirror the adverse metabolic conditions (e.g., increased gut permeability) [33]. In the present study, the Shannon index increased on day 21 of lactation compared with on day 105 of pregnancy, which is consistent with previous studies [12, 34]. These findings suggested that the gut microbiota might have a positive effect on sow's metabolism during lactation. In addition, bacterial community composition analysis (*β*-diversity) showed that the gut microbial composition of sows was significantly altered during pregnancy and lactation, which might be caused by delivery, different diets, or the change of feeding environment.

The dominant phyla were *Firmicutes* and *Bacteroides* in animal gut [35] and the relative abundances of which were higher than other microbiota in our study, though *Firmicutes* and *Bacteroidetes* relative abundances were not significantly different during pregnancy and lactation. Interestingly, we also found that several gut microbiota composition varied from pregnancy to lactation stages. *Streptococcus* (*Firmicutes* phylum), *Treponema* (*Spirochaetes* phylum), and *Spirochaetes* relative abundances were significantly reduced, but *Desulfovibrio* relative abundance was increased during lactation, relative to several pregnancy stages in this study. These alterations might be influenced by dietary fiber from feed ingredients in the present study. Previous study found that *Streptococcus* decreased while *Desulfo*vibrio increased in overweight and obese pregnant women after ingested high fiber diets, though the changed fiber had no effects on microbial alpha diversity [36], which is not consistent with our findings. In addition, *Streptococcus*, the dominant human milk bacteria [37], was thought to induce lactation mastitis [38]. Previous studies showed that decreased *Spirochaetes* abundance relieved constipation in mini sows [39] and that *Spirochaetes* abundance was higher in obese pigs compared with lean pigs [40]. Thus, these findings suggested that sows at different pregnancy and lactation stages show varied intestinal microbiota composition, which may depend on host metabolism at disparate physiological states.

Metabolites produced by gut microbiota govern host metabolism and health [41]. Several studies have shown that SCFAs, particularly butyrate, produced by the *Firmicutes* using dietary fiber and resistant starch as the substrates, mainly provide energy for host colonic epithelium cells [42], exert relieving metabolic syndrome effects (e.g., anti-Type 2 diabetes during pregnancy) [43], and prevent hypertension occurrence and/or development during pregnancy [44]. In the present study, the SCFAs (including acetate, butyrate, and valerate) levels decreased during pregnancy than those during lactation and presented a decreasing trend from early pregnancy to late pregnancy, suggesting that the SCFAs not only favored maternal energy requirement but were absorbed to benefit fetuses' growth and development [45]. These alterations in intestine SCFA levels might be due to the stronger absorption capacity in Bama mini pigs during late pregnancy, and the specific reasons remain to be further investigated. Besides, the BCFAs are mainly derived from the catabolism of proteins in the intestinal lumen, which are the products of oxidative deamination of leucine, isoleucine, and valine [46]. The present study showed that isobutyrate, isovalerate, and total BCFA levels were higher during lactation than pregnancy, which might be due to the increased feed intake (2.4 kg per day) and CP level (16.30%) in lactation diets, thus increasing the catabolism of these amino acids in the hindgut [12]. However, the physiological significance of which remains to be further explored.

In addition, polyamines, including putrescine, spermine, and spermidine, present functions in gene expression regulation, DNA and protein synthesis, and cell proliferation and differentiation [47]. In the present study, both putrescine and spermidine levels increased during pregnancy compared with lactation, indicating that they are made available for rapid fetus growth in the second and third trimesters, since polyamines play a key regulatory role in angiogenesis, as well as placental and fetal growth and development [48]. Indole and skatole in the hindgut are produced by the decomposition of proteins undigested in the small intestine [49]. However, the present study showed that indole and skatole levels were decreased during pregnancy but were increased during lactation, which might result from undigested protein metabolites in lactation sows, because the dietary crude protein level during lactation (16.30%) was higher than pregnancy (12.82%). A previous study also showed that high wheat bran-containing diets could affect gut microbiota structure in pregnant sows [50], suggesting that maternal dietary composition might influence gut microbial metabolites by altering microbiota composition.

Metabolic syndromes, including hyperglycemia and excess energy intake, can be induced by the high plasma TG, TC, LDL-C, or GLU levels [51]. Previous study showed that *Akkermansia muciniphila* could enhance the glucose tolerance, regulating host's metabolism [52]. In the present study, the GLU level and *Akkermansia* abundance were the highest on day 7 of lactation, which is consistent with previous findings [53]. Recent studies showed that *Akkermansia* abundance reduced in host with type 2 diabetes [25, 54], which was negatively correlated with the metabolic syndromes, obesity, type 2 diabetes, and hypertension [55]. It was also reported that *Akkermansia* could play an immune-modulatory role by interacting with host intestinal epithelial cells [56]. Hence, it is interesting to investigate the relationship between intestinal microbiota and hormones or metabolites levels in pregnant and lactating sows. Here, we showed a positive correlation between TG and CHE levels, and *Akkermansia* and *Streptococcus* relative abundances. *Akkermansia* abundance was also positively correlated with spermidine level but negatively with LH, FSH, PRL, butyrate, or skatole levels. These relationships implied that there might be a synergistic effect or negative feedback mechanism between fecal microbiota and their metabolites and/or plasma metabolites.

In conclusion, gut microbiota was mainly dominated by the *Firmicutes*, *Bacteroidetes*, and *Spirochaetes* phyla, though the *Firmicutes* and *Bacteroidetes* phyla showed no significant differences during pregnancy and lactation. Compared with pregnancy, the fecal microbial alpha diversity and relative abundances of *Spirochaetes*, *Streptococcus*, and *Treponema* decreased during lactation. *Akkermansia* relative abundance was the highest on day 7 of lactation. In addition, there is a tight cross-talk between gut microbial remodeling and host physiological status, including plasma biochemical parameter and reproductive hormone changes, as well as fecal metabolite changes. Further, it will be interesting to explore whether and how *Akkermansia* influences pregnancy and lactation in mammals.

## Figures and Tables

**Table 1:** Composition and nutrient levels of the basal diets (air-dry basis; %).

Items	Pregnant sows' diet	Lactating sows' diet
Ingredients		
Corn	37.50	66.00
Soybean meal	9.50	25.00
Wheat bran	14.00	5.00
Barley	25.00	
Soybean hull	10.00	
Pregnant sows' premix^1^	4.00	
Lactating sows' premix^2^		4.00
Total	100.00	100.00
Nutrient levels^3^		
DE (MJ/kg)	12.55	13.87
CP	12.82	16.30
CF	4.56	2.87
SID Lys	0.48	0.75
SID met+Cys	0.43	0.51
SID Thr	0.37	0.53
SID Trp	0.13	0.17
Ca	0.62	0.65
P	0.47	0.50

Note: ^1^Pregnant sows' premix provided the following per kg of diets: CaHPO_4_·2H_2_O 10 g, NaCl 4 g, CuSO_4_·5H_2_O 80 mg, FeSO_4_·H_2_O 360 mg, ZnSO_4_·H_2_O 240 mg, MnSO_4_·H_2_O 100 mg, MgSO_4_·7H_2_O 1 g, 1% ICl 50 mg, 1% Na_2_SeO_3_ 36 mg, 1% CoCl_2_ 16 mg, NaHCO_3_ 1.4 g, VA 10000 IU, VD_3_ 1800 IU, VE 20 mg, VK_3_ 2.4 mg, VB_1_ 1.6 mg, VB_2_ 6 mg, VB_6_ 1.6 mg, VB_12_ 0.024 mg, folic acid 1.2 mg, nicotinamide 20 mg, pantothenic acid 12 mg, biotin 0.12 mg, ferrous glycinate 100 mg, choline chloride 1 g, phytase 200 mg, fruity 80 mg, and limestone 12 g. ^2^Lactating sows' premix provided the following per kg of the diet: CaHPO_4_·2H_2_O 10 g, NaCl 4 g, CuSO_4_·5H_2_O 80 mg, FeSO_4_·H_2_O 360 mg, ZnSO_4_·H_2_O 240 mg, MnSO_4_·H_2_O 100 mg, 1% ICl 50 mg, 1% Na_2_SeO_3_ 36 mg, 1% CoCl_2_ 16 mg, NaHCO_3_ 1.4 g, VA 10000 IU, VD_3_ 1800 IU, VE 20 mg, VK_3_ 2.4 mg, VB_1_ 1.6 mg, VB_2_ 6 mg, VB_6_ 1.6 mg, VB_12_ 0.024 mg, folic acid 1.2 mg, nicotinamide 20 mg, pantothenic acid 12 mg, biotin 0.12 mg, lysine 1.5 g, ferrous glycinate 100 mg, choline chloride 1 g, phytase 200 mg, fruity 80 mg, and limestone 12 g. ^3^Nutrient levels were calculated values. Ca: calcium; CF: crude fiber; CP: crude protein; DE: digestible energy; P: phosphorus; SID: standard ileum digestible.

## Data Availability

The data used to support the findings of this study are available from the corresponding author upon request.

## Supplementary Materials

Supplementary Materials Table S1: mean count of clean sequences and OTUs among five stages of gestation and lactation.
