# Supplementary material for: Alterations in the Blood Parameters and Fecal Microbiota and Metabolites during Pregnant and Lactating Stages in Bama Mini Pigs as a Model
Source: Mediators Inflamm. 2020 Oct 26;2020:8829072. doi: 10.1155/2020/8829072 (PMC7607286; doi:10.1155/2020/8829072)
Supplement: Supplementary Materials — Table S1: mean count of clean sequences and OTUs among five stages of gestation and lactation. [file 8829072.f1.docx]

Alterations in the blood parameters and fecal microbiota and metabolites during pregnant and lactating stages in Bama-mini pigs as a model

Ma Cui ^1,2^, Gao Qian Kun ^1^, Zhang Wang Hong ^1,2^, Md. Abul Kalam Azad^1^, and Kong Xiang Feng ^1*^

*^1^ CAS Key Laboratory of Agro-ecological Processes in Subtropical Region, Hunan Provincial Key Laboratory of Animal Nutritional Physiology and Metabolic Process, National Engineering Laboratory for Pollution Control and Waste Utilization in Livestock and Poultry Production, Institute of Subtropical Agriculture, Chinese Academy of Sciences, Changsha 410125, Hunan, China*

*^2^ University of Chinese Academy of Sciences, Beijing 100008, China*

***** Correspondence should be addressed to Xiangfeng Kong; nnkxf@isa.ac.cn

**Table S1 Mean count of clean sequences and OTUs among five stages of gestation and lactation**

| Items | P45 | P75 | P105 | L7 | L21 |
| --- | --- | --- | --- | --- | --- |
| Effective sequences | 33501 | 41103.5 | 44598 | 39259 | 39429 |
| OTUs | 1431 | 1565 | 1246 | 972 | 1264 |

Note: P45, P75, and P105 represent the days 45, 75, and 105 of pregnancy, respectively. L7 and L21 represent the day 7 and day 21 of lactation, respectively. OUTs: operational taxonomic units.
